# Pathogenesis of Noroviruses, Emerging RNA Viruses

**DOI:** 10.3390/v2030748

**Published:** 2010-03-23

**Authors:** Stephanie M. Karst

**Affiliations:** Center for Molecular and Tumor Virology, Department of Microbiology and Immunology, Louisiana State University Health Sciences Center, Shreveport, Louisiana 71130-3932, USA; E-Mail: skarst@lsuhsc.edu; Tel.: +1-318-675-8122; Fax: +1-318-675-5764

**Keywords:** norovirus, calicivirus, pathogenesis

## Abstract

Human noroviruses in the family *Caliciviridae* are a major cause of epidemic gastroenteritis. They are responsible for at least 95% of viral outbreaks and over 50% of all outbreaks worldwide. Transmission of these highly infectious plus-stranded RNA viruses occurs primarily through contaminated food or water, but also through person-to-person contact and exposure to fomites. Norovirus infections are typically acute and self-limited. However, disease can be much more severe and prolonged in infants, elderly, and immunocompromised individuals. Norovirus outbreaks frequently occur in semi-closed communities such as nursing homes, military settings, schools, hospitals, cruise ships, and disaster relief situations. Noroviruses are classified as Category B biodefense agents because they are highly contagious, extremely stable in the environment, resistant to common disinfectants, and associated with debilitating illness. The number of reported norovirus outbreaks has risen sharply since 2002 suggesting the emergence of more infectious strains. There has also been increased recognition that noroviruses are important causes of childhood hospitalization. Moreover, noroviruses have recently been associated with multiple clinical outcomes other than gastroenteritis. It is unclear whether these new observations are due to improved norovirus diagnostics or to the emergence of more virulent norovirus strains. Regardless, it is clear that human noroviruses cause considerable morbidity worldwide, have significant economic impact, and are clinically important emerging pathogens. Despite the impact of human norovirus-induced disease and the potential for emergence of highly virulent strains, the pathogenic features of infection are not well understood due to the lack of a cell culture system and previous lack of animal models. This review summarizes the current understanding of norovirus pathogenesis from the histological to the molecular level, including contributions from new model systems.

## 1. Introduction

**Figure 1:**
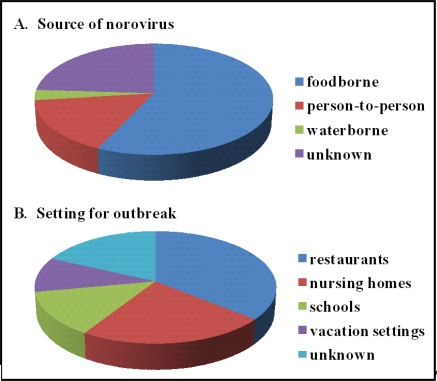
Norovirus outbreak characteristics. The Centers for Disease Control collected data from 232 norovirus outbreaks between July 1997 and June 2000. The results of this surveillance are summarized here for the source of virus (**A**) and the type of setting affected by the outbreak (**B**). Data are reproduced from http://www.cdc.gov/ncidod/dvrd/revb/gastro/norovirus-factsheet.htm.

Noroviruses constitute a genus within the family *Caliciviridae*. The human pathogens within this genus cause at least 95% of nonbacterial gastroenteritis outbreaks, and 50% of all gastroenteritis outbreaks, throughout the world. It is estimated that there are approximately 23 million norovirus infections per year in the United States alone, causing 50,000 hospitalizations and 300 deaths [1]. Based on a recent review of diagnostic studies testing for norovirus infections in clinical settings, it is estimated that they cause over one million hospitalizations and 200,000 deaths in young children in developing countries annually [2]. Noroviruses are highly transmissible and can spread via exposure to contaminated food or water sources, person-to-person contact, aerosolized vomitus particles, and fomites (Figure 1A). They are considered by CDC to be the most common cause of foodborne disease outbreaks [3]. Numerous features of norovirus infections have led to their classification as Category B biodefense agents, including their high infectivity, extreme stability, resistance to common disinfectants, and ability to cause incapacitating disease. Persons of all age groups are susceptible to infection and secondary infections are common [4–6]. In recent years, there has been a dramatic increase in the number of reported norovirus outbreaks. These emerging RNA viruses have historically been very difficult to study but numerous advances in the field have begun to contribute to the overall understanding of norovirus pathogenesis.

## 2. Clinical Disease and Epidemiology

### 2.1. Clinical Disease

In immunocompetent adults, the course of norovirus infection is rapid, with an incubation period of 24–48 hours and resolution of symptoms within 12–72 hours [7]. Symptoms include vomiting and diarrhea with or without nausea and abdominal cramps. Low-grade fever and malaise can also develop. While norovirus infection typically causes an acute bout of gastroenteritis that resolves within days of the onset of symptoms, norovirus-induced disease can be much more severe and prolonged in specific risk groups. For example, infants and young children can develop more severe gastroenteritis following norovirus infection, with symptoms lasting up to six weeks [8–11]. Noroviruses have been reported to be second only to rotaviruses in causing severe childhood gastroenteritis [10,12]; considering the recent success in vaccinating infants against rotavirus infections, noroviruses will likely become the most common cause of childhood diarrheal disease in the foreseeable future. In addition, prolonged symptomatic infection has been well-documented in transplant patients and other immunosuppressed individuals, with symptoms lasting over two years [13–19]. Similarly, norovirus infection can be particularly severe in the elderly, even resulting in death [20–23]. One recent study also reported that patients with inflammatory bowel disease may present with bloody diarrhea upon norovirus infection [24].

The complete scope of norovirus-induced morbidity worldwide and particularly in developing nations has been difficult to ascertain due to several factors (discussed in more detail elsewhere [20,25]). First, norovirus detection is difficult because these viruses cannot be propagated in cell culture and they are genetically variable, complicating RT-PCR-based detection assays. Second, there is a lack of reporting to health officials because of the acute nature of disease. Finally, national and international diagnostic/surveillance programs are not standardized, if present at all. Importantly though, there has recently been increased recognition of the burden of norovirus disease due to significant improvements in norovirus diagnostic assays and an increased awareness of the need for surveillance standardization. Along these lines, a global surveillance network called NoroNet (http://www.noronet.nl/noronet/) was recently established that aims to monitor norovirus epidemics in an effort to limit their scope. Continued efforts of this sort will be critical to delineate the global burden of norovirus disease.

As norovirus diagnostics have improved, there have been numerous reports of norovirus associations with clinical outcomes other than gastroenteritis. For example, one case report detected norovirus RNA in the serum and cerebrospinal fluid of a child with encephalopathy [26]. In addition, during a norovirus outbreak among military personnel in Afghanistan in May 2002, three infected patients presented with gastroenteritis, diminished alertness, headache, neck stiffness, and light sensitivity; one of these patients also displayed disseminated intravascular coagulation [27]. Noroviruses have also been implicated in necrotizing enterocolitis in premature infants [28], postinfectious irritable bowel syndrome [29], and benign infantile seizures [30,31]. While these case reports provide only anecdotal evidence that norovirus infection can have varied clinical outcomes, they do suggest that noroviruses should be considered as potential etiological agents of diseases other than gastroenteritis.

### 2.2. Genetic Diversity

Noroviruses are classified in five genogroups (GI-GV), three of which (GI, GII, and GIV) contain primarily human viruses [32,33]. Multiple porcine noroviruses have been placed in GII [34,35]. Genogroup III (GIII) contains bovine noroviruses [36,37] and GV contains murine noroviruses [38–42]. The genogroups are further subdivided into 31 distinct clusters or genotypes (8 GI, 19 GII, 2 GIII, 1 GIV, and 1 GV genotypes) [4,32]. Norovirus strains are typically named after the location in which they were first identified; e.g., the Farmington Hills strain was first shown to be responsible for gastroenteritis cases in Farmington Hills, Michigan [43]. They are also commonly referred to by their genogroup and genotype; e.g., the Farmington Hills strain is a GII.4 (genogroup II, genotype 4) strain. Noroviruses display a wide degree of genetic variability – members within a genogroup differ by 45–61% in their capsid genes, members within a genotype differ by 14–44%, and strains within a genotype differ by 0–14% [32]. This amount of intra-genus variation is high even compared to genera of other plus-strand RNA virus families [32]. This high degree of variability is undoubtedly one factor that complicates protective norovirus immunity.

### 2.3. Epidemiology

Norovirus outbreaks occur most commonly in semi-closed communities such as nursing homes, schools, hospitals, cruise ships, disaster relief/evacuation sites, and military settings (Figure 1B) [33,44–47]. Nosocomial norovirus outbreaks are one of the most common causes of hospital ward closures [48]. Outbreaks display wintertime seasonality, explaining why norovirus disease is referred to as the winter vomiting disease [49]. Several factors most likely contribute to the explosive nature of norovirus outbreaks, including the high infectivity of norovirus particles [50], the persistence of noroviruses in the environment [51–53], prolonged shedding of virus from both symptomatic and asymptomatic individuals [54–56], and a lack of lasting immunity [4–6]. In 2002, a sharp rise in norovirus outbreaks worldwide was recognized which correlated with the emergence of a new virus variant, specifically a GII.4 norovirus strain [51,57]. Interestingly, researchers have determined that GII.4 strains resulted in pandemics in 1995–1996 (US95/96 strain) [33,58,59], 2002 (Farmington Hills strain) [43], 2004 (Hunter strain) [60], and 2006 (Minerva/2006b and Laurens/2006a strains) [61]. These GII.4 variants spread rapidly and globally and are thought to account for 70–80% of all norovirus outbreaks at least since 2002 [62]. A recent study demonstrates that GII.4 strains associated with severe illness were circulating as early as 1974, and that the ancestral strain most likely emerged in the 1960s [63]. It is unclear why the GII.4 norovirus strains are so predominant but possibilities include increased environmental stability, transmissibility, and virulence [64].

While it is widely accepted that noroviruses cause a majority of epidemic gastroenteritis worldwide, they have not received significant attention as a cause of sporadic gastroenteritis. However, improved diagnostics has shed light on the unappreciated breadth of norovirus infection in this setting [12]. Based on numerous studies around the world, it is now recognized that noroviruses are an important cause of illness in hospitalized children (discussed in detail in several recent reviews [12,25,65]), second to or as prevalent as rotavirus infections. They are also a previously unappreciated cause of traveler’s diarrhea [66–68].

## 3. Pathophysiology

Because of the lack of a cell culture system and the historical lack of animal models of norovirus infection, the extent of our knowledge regarding pathogenesis of norovirus infection comes primarily from physical, histological, and biochemical studies of infected human volunteers. In recent years, work in porcine, bovine, and murine models has also begun to contribute to our understanding of norovirus pathogenesis.

### 3.1. Histological Alterations in the Intestine

Histological analysis of proximal intestinal biopsy samples from human volunteers that become ill after administration of either a GI (Norwalk; GI.1) or GII (Hawaii; GII.1) norovirus demonstrate an intact intestinal mucosa with specific histological changes, including broadening and blunting of the villi, shortening of the microvilli, enlarged and pale mitochondria, increased cytoplasmic vacuolization, and intercellular edema [69–72]. While abnormalities are apparent in intestinal epithelial cells of norovirus-infected volunteers, electron microscopy analysis reveals that these cells remain intact [69,70]. Crypt cell hyperplasia has also been reported following norovirus infection [71,73]. Only proximal intestinal biopsies from infected individuals were obtained in early volunteer studies so it remains to be determined whether the distal intestine is also affected by norovirus infection. In addition to enterocyte changes, norovirus infection results in a mild inflammatory infiltration into the lamina propria that has been observed in humans infected with the Norwalk [69,71] and Hawaii viruses [70,72], gnotobiotic calves infected with the human GII.4 norovirus HS66 [74], and mice infected with murine norovirus 1 (MNV-1) [75]. A recent study also reports an increased number of intraepithelial cytotoxic T cells in the duodenum of norovirus-infected patients 0–6 days after symptom onset [73]. While specific intestinal lesions are observed during the time of norovirus illness, they completely resolve within two weeks.

Several recent studies suggest that noroviruses cause apoptosis of enterocytes in humans [73], pigs [76], and mice [75]. It is unclear whether viral infection of enterocytes directly induces apoptosis or whether a viral component secreted from other cells acts upon bystander enterocytes to induce their programmed cell death. Murine noroviruses and feline caliciviruses have been demonstrated to cause apoptosis of infected macrophages and epithelial cells, respectively, *in vitro* through the mitochondrial pathway [77–81], suggesting that apoptosis *in vivo* may be due to direct effects of infection. Troeger *et al.* postulate that the observed influx of intraepithelial CD8^+^ lymphocytes during norovirus infection could cause enterocyte apoptosis upon perforin release [73]. Thus, it is possible that both direct and indirect mechanisms contribute to norovirus-induced apoptosis of enterocytes.

### 3.2. Physical and Biochemical Manifestations

In one human volunteer study, it was observed that norovirus infection causes a marked delay in gastric emptying thought to be related to the high incidence of vomiting episodes during norovirus infections [82]. Such a pathophysiologic outcome to infection could be caused by an alteration of gastric motor functions or inflammation of the pyloric junction between the stomach and intestine. While mice lack an emetic reflex and thus do not vomit, it has been demonstrated that STAT1^−/−^ mice develop dramatic gastric bloating upon MNV-1 infection [75]. The recapitulation of delayed gastric emptying in a small animal model should facilitate a mechanistic dissection of the basis of this outcome of norovirus infection. A transient malabsorption of D-xylose, fat, and lactose also occurs during acute norovirus infection [71,83]. This malabsorption correlates with shortened microvilli and decreased activity of specific brush border enzymes on enterocytes, including alkaline phosphatase, sucrase, trehalase, and possibly lactase [69]. It is presently unclear whether these changes are due to direct virus-mediated damage to enterocytes, virus-induced alterations of brush border enzyme expression levels (as has been noted for rotavirus [84]), or bystander/immunopathologic effects. Moreover, it is unknown whether there is also a secretory component to norovirus-induced diarrhea.

### 3.3. Systemic Infection

While it has long been assumed that norovirus infection is confined to the intestine, there is no direct proof for this claim and several recent findings suggest that this dogma be re-considered. First, a recent study detected norovirus RNA in the serum of 15% of infected individuals [85]. Second, work in animal models of norovirus infection support virus dissemination past the intestine: (i) a transient viremia was detected in 50% of gnotobiotic pigs infected with HS66 [76]; (ii) one of five HS66-infected gnotobiotic calves had detectable viral RNA in their serum [74]; and (iii) murine noroviruses are well-documented to spread to mesenteric lymph nodes (MLNs) that drain from the intestine [41,75,86–89] and to peripheral tissues [41,75,86,88,89]. In particular, MNV-1 replicates efficiently in the spleen and induces specific splenic histological changes, including activation of cells in the white pulp and hypertrophy of cells in the red pulp [75]. The physiological relevance of these splenic changes is unclear. The possibility that human noroviruses disseminate to peripheral sites is a clinically relevant question since mild or sporadic pathologies associated with human norovirus infection of peripheral tissues may have been missed due to the difficulties in their detection and the assumption that they are limited to the intestinal tract. Supporting this idea, there have been several recent associations between norovirus infection and extraintestinal pathologies including encephalopathy [26], disseminated intravascular coagulation [27], and benign infantile convulsions [30,31]. Interestingly, rotaviruses have also recently been confirmed to spread to peripheral tissues and cause histological changes at these sites [90–92]. The mechanism of norovirus dissemination is unknown but at least the murine noroviruses infect dendritic cells (DCs), a cell type known to actively migrate from tissues to draining lymph nodes. Thus, it is possible that noroviruses utilize DC infection to facilitate their extraintestinal spread.

### 3.4. The Course of Norovirus Infection

Norovirus infections are typically considered to be short-lived, lasting for only a few days; however, increasing evidence points to prolonged (or perhaps persistent) infections in some scenarios. Although the symptoms caused by human norovirus infection typically resolve within several days, virus particles can be shed from asymptomatic individuals for weeks after exposure [54–56,93]. Further, symptomatic infection has been documented in immunosuppressed individuals with symptoms lasting for over 2 years [13–15] and in immunocompetent children with symptoms lasting for up to 6 weeks [9,56]. Additionally, pigtail macaques infected with a human norovirus shed virus in their feces for at least 3 weeks [94]. It is also clear that murine noroviruses can infect wild-type hosts for weeks [41,86,87]. Interestingly, virus is detected not only in fecal samples of these mice but also in MLNs and in some cases intestines and spleens. Thus, there may be mucosal and extraintestinal sites of prolonged norovirus infection. Finally, it has long been recognized that feline caliciviruses establish chronic infections in cats, with animals continuing to shed virus for at least one month following infection and some even becoming life-long carriers [95]. Because these carrier cats are contagious, they represent an important reservoir for the maintenance of feline calicivirus in the feline population [96,97]. Overall, noroviruses can infect their hosts for weeks or months even in the presence of a fully functional immune system. Similar to the situation with feline caliciviruses, it is possible that individuals experiencing prolonged human norovirus infection act as reservoirs of virus, perpetuating its spread within a population.

The reason(s) for prolonged norovirus infection is currently unclear but mechanisms underlying the ability of other viruses to establish persistence may logically play a role. A fundamental feature of persistent viral infections is that the host immune response is ineffective at completely clearing infectious virus [98]. Consistent with this, adaptive immune cells are critical to limit the magnitude of norovirus infection since mice lacking B and T cells contain extremely high levels of MNV-1 within numerous tissues and feces for months following infection [39,99]. The inability to clear persistent infection with other viruses is commonly associated with viral evasion. For example, in persistent viruses such as hepatitis C virus (HCV) there is commonly an alteration of the antigenic epitopes encoded by the virus. Similarly, it has been demonstrated that the feline calicivirus genome acquires mutations in areas predicted to be important for immune recognition during chronic infection, suggesting that altered antigenicity plays a role in maintaining calicivirus persistence [100,101]. One study reports a similar accumulation of mutations within the hypervariable P2 capsid domain in a patient chronically infected with a human norovirus [15]. Interestingly, there is evidence for feline calicivirus that such progressive evolution of a single virus within a host accounts for only a minority of chronic infections; the majority appears to be instead due to cycles of sequential reinfection with either a variant of the same virus strain or with a different strain followed by a period of progressive evolution [102].

Persistent viral infections are also commonly associated with impairment of the normal functioning of immune cells upon infection. Although there is currently no direct evidence that noroviruses impair immune function, it is interesting to note that murine noroviruses infect DCs [103] since many persistent viruses target this cell type and specifically impair their ability to activate T and B cells [98]. Overall, the mechanisms by which noroviruses maintain prolonged infection remain incompletely understood, but they may play a critical role in impairing adaptive immune responses such that they fail to protect from secondary challenge. It will be interesting in future studies to examine the relationship between prolonged norovirus infection and the lack of protective immunity elicited by primary infection. Prolonged virus shedding from individuals in whom symptomatic infection has resolved also surely contributes to the difficulty in controlling norovirus outbreaks.

## 4. Cellular Determinants of Norovirus Pathogenesis

The precise cell tropism of human noroviruses is unknown. When intestinal biopsy sections from volunteers infected with either a GI.1 or a GII.1 norovirus were analyzed by electron microscopy, virus particles were not observed [70,83], leading investigators to postulate that human noroviruses infect very few cells *in vivo.* This hypothesis is supported by work in animal models (described below). Furthermore, numerous attempts to identify a relevant cell culture system for these viruses have been unsuccessful [104]. The inability to propagate human noroviruses has long been the major obstacle to studying this virus family. However, numerous recent advances in this field offer promise of overcoming this barrier. Moreover, recent work in animal models suggests that multiple intestinal cell types including enterocytes, macrophages (Mφs), and DCs may support at least low level *in vivo* replication.

### 4.1. Recent Advances of In Vitro Human Norovirus Infection Models

Extensive efforts have failed to detect norovirus replication in intestinal epithelial cell lines [104]. There has been a single report describing human norovirus infection of a 3-dimensional model of human small intestinal epithelium [105]; however, several independent laboratories failed to detect increased viral genome in these cells during the period of noted cytotoxicity, strongly suggesting that cellular pathology was not due to viral replication [106]. Based on the tropism of murine noroviruses for DCs and Mφs [103], *in vitro* infections of human DCs and Mφs derived from monocytes isolated from peripheral blood mononuclear cells (PBMCs), or myeloid DCs directly isolated from PBMCs, were performed with the G1.1 Norwalk virus. There was no evidence for viral replication in these cells (Lay MK, Atmar RL, Guix S, Bharadwaj U, He H, Neill FH, Sastry JK, Yao Q and Estes MK, manuscript in preparation). While these results demonstrate that at least one human norovirus does not infect peripheral DCs and Mφs *in vitro*, DCs and Mφs can differ significantly in pathogen susceptibility depending on their source and differentiation [107–109]; thus it remains possible that other types of DCs and Mφs (*i.e.,* mucosal cells) are permissive to human noroviruses.

Based on several recent studies, it is now clear that the block to human norovirus propagation *in vitro* occurs at the level of receptor binding, internalization, or genome uncoating. Specifically, Guix *et al.* demonstrated that transfecting norovirus RNA into Huh-7 cells results in a complete single round of viral replication culminating in progeny virion release [110]. Furthermore, transfection of nonpermissive cells with infectious norovirus clones driven by either the T7 polymerase or the host RNA polymerase II results in genome replication and virion production [111–114]. Similarly, a norovirus replicon has been generated that results in genome replication and nonstructural protein production in stably transfected cells [115]. Overall, it can be concluded that bypassing the initial steps of virus entry and uncoating overcomes the block to norovirus replication in cultured cells.

### 4.2. Human Norovirus Cell Tropism in Animal Models

**Figure 2:**
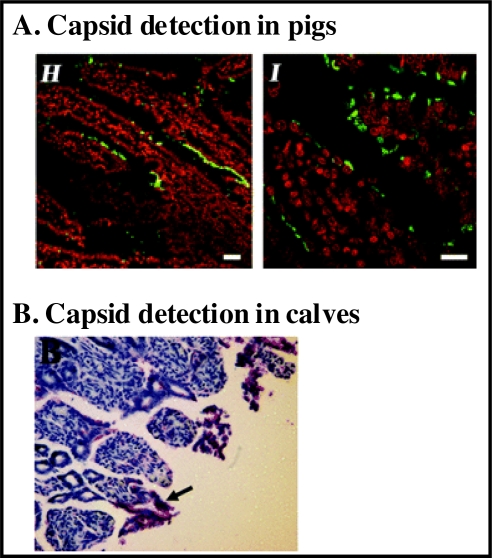
The intestinal cell tropism of HS66 in gnotobiotic pigs and calves. (**A**) Paraffin-embedded sections of the duodenum of HS66-infected gnotobiotic pigs prepared 4 dpi were stained with a monoclonal antibody against the GII human norovirus capsid protein (called NS14). Fluorescently conjugated anti-mouse secondary antibody facilitated visualization of capsid through immunofluorescent confocal microscopy (in green). Nuclei were stained with propidium iodide (in red). Data are reproduced from *J. Virol.* **2006**, *80*, 10372–10381. (**B**) Paraffin-embedded sections of the jejunum of HS66-infected gnotobiotic calves prepared 3 dpi were stained with NS14. Viral capsid was visualized through immunohistochemistry. Data are reproduced from *J. Virol.* **2008**, *82*, 1777–1786.

Both gnotobiotic pigs and gnotobiotic calves are susceptible to infection with at least one human norovirus, a GII.4 virus called HS66 [74,76]. Infected pigs and calves develop diarrhea and histopathological changes are evident in the intestines of infected calves. In HS66-infected gnotobiotic pigs, virus particles are apparent in cytoplasmic vesicles within enterocytes [76]. Moreover, viral capsid can be detected in enterocytes located at villous tips and along the sides of villi, but rarely in crypts, of the duodenum and jejunum (Figure 2A). Nonstructural protein indicative of viral replication can also be detected in a limited number of enterocytes. In HS66-infected gnotobiotic calves, viral structural protein is detectable in enterocytes (Figure 2B) and in lamina propria cells resembling Mφs along the jejunum and ileum, but not along the duodenum [74]. The discrepancy between these animal model studies and earlier studies that failed to detect virus in human intestinal sections could be due to (i) virus strain-dependent differences (GII.4 *versus* GI.1 and GII.1 viruses, respectively); (ii) virus dose differences; (iii) species-specific disparities in norovirus infection; or (iv) differences in assay sensitivity between studies.

### 4.3. Murine Norovirus Cell Tropism

**Figure 3:**
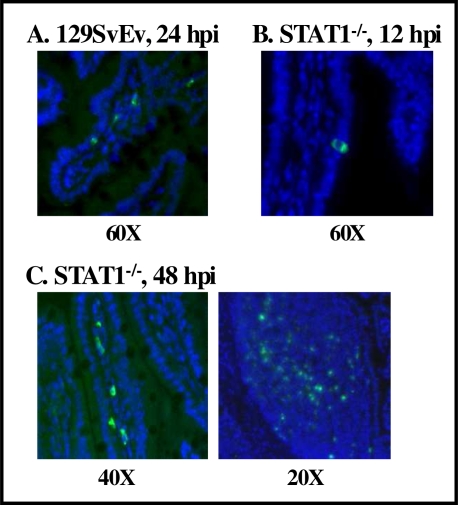
The intestinal cell tropism of MNV-1 in wild-type and STAT1^−/−^ mice. Intestinal sections prepared from MNV-1-infected 129SvEv (**A**) or STAT1^−/−^ (**B** and **C**) mice were stained with guinea pig polyclonal antibody raised against the MNV-1 Pro:Pol nonstructural protein. Fluorescently conjugated secondary antibody was then used to visualize viral protein through immunofluoresence, in which viral protein is pseudocolored in green and nuclei in blue. Data are reproduced from *J. Virol.* **2007**, *81*, 3251–3262.

A number of murine norovirus strains have now been isolated from mouse colonies in academic research settings around the world [39–42,86]. Murine noroviruses are enteric in nature: (i) most strains were originally isolated from fecal samples or MLNs; (ii) they are infectious orally and can be detected in mucosal sites (intestines, MLNs) during infection; and (iii) they are shed in feces [39–42,75,86]. At least one murine norovirus causes decreased fecal consistency and mild intestinal inflammation in infected wild-type mice, and severe gastroenteritis in STAT1^−/−^ mice [75,88]. Murine noroviruses are known to replicate in DCs and Mφs, but not other cell types, *in vitro* [39–41,86,103,116,117]. Moreover, in MNV-1-infected wild-type mice, viral nonstructural protein can be detected in lamina propria cells morphologically resembling DCs and Mφs of rare intestinal villi at 24–48 hpi (Figure 3A) [75]. The *in vivo* infection of DCs and Mφs by MNV-1 is further supported by the immunohistochemical detection of MNV-1 antigen in cells that resemble Mφs in the liver, and Mφs and DCs in the spleen [103]. It has also been reported that immunodeficient mice naturally infected with a murine norovirus contain viral antigen in the cytoplasm of inflammatory cells in the liver, splenic red and white pulp, intestinal lamina propria, intestinal lymphoid follicles, lung, pleural and peritoneal cavities, and MLNs [89,118]. In the absence of an intact interferon signaling pathway, MNV-1 antigen is detected more abundantly and in additional intestinal cell types of infected mice. Specifically, viral antigen can be detected in enterocytes of STAT1^−/−^ mice at 12 hpi (Figure 3B), and lamina propria and Peyer’s patch cells at 24–48 hpi (Figure 3C) [75]. Thus, interferon may restrict the cell tropism of noroviruses, as has been shown for a number of other viruses [119–121]. If this is the case, norovirus strains with differing sensitivity to interferon responses may display disparate cell tropism.

### 4.4. Norovirus Receptors

Human noroviruses recognize histo-blood group antigens (HBGAs) that are expressed on the surface of mucosal epithelial cells [122]. HBGAs are neutral carbohydrates linked to proteins or lipids on cell surfaces. The glycosyltransferases that control their synthesis are encoded by the highly polymorphic ABO, Lewis, and secretor gene families. The association of noroviruses with HBGAs has been demonstrated to be essential for certain virus strains. This is best exemplified by the strict correlation between secretor status and susceptibility to Norwalk virus infection – Norwalk virus recognizes α1,2-linked fucose residues [63] whose expression on gut epithelial cells and in body fluids is dependent on a wild-type *FUT2* gene; while individuals with a wild-type *FUT2* gene (∼80% of the population; referred to as secretors) are susceptible to Norwalk virus infection, individuals that contain a null *FUT2* allele (∼20% of the population; referred to as nonsecretors) are completely resistant [123].

**Figure 4:**
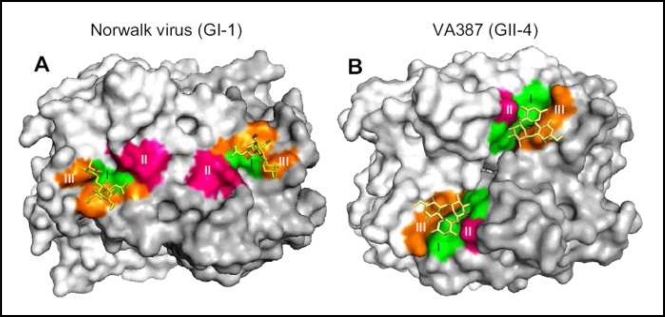
The HBGA binding interface on norovirus particles is genogroup-specific. Crystal structures have been determined for P dimers of a representative GI norovirus (**A**) and a representative GII norovirus (**B**) complexed with HBGA. Surface models are shown here, with P dimers colored gray (one monomer is darker gray than the other) and the three major components of the HBGA binding interface colored green, red, and orange. The HBGA is colored yellow. While the binding sites on both viruses lie within the exposed P2 domain, the exact residues and HBGA binding mode differ. Moreover, note that the HBGA binding interface of Norwalk virus is located within a single monomer while the interface of VA387 spans both monomers. Data are reproduced from *PLoS ONE* **2009**, *4*, e5058.

A number of distinct norovirus strain-specific binding profiles have been reported [124–126]. As described recently by Tan *et al.*, strains can be segregated generally into those that bind A/B epitopes and those that bind Lewis epitopes [127]. While GI and GII strains are contained within both binding categories, there are genogroup-specific differences in the receptor binding interface of the norovirus capsid that interacts with the HBGA. Thus, even if a GI and a GII norovirus interact with the same HBGA, the precise residues involved in this binding will most likely be different (Figure 4). In terms of the highly prevalent GII.4 noroviruses, strains circulating in 1974, 1977, and 1987 all display a common HBGA binding pattern (H type 3, Lewis^Y^, and B antigens); a strain circulating in 1997 binds the same set of HBGAs in addition to A and Lewis^B^ antigens; a strain isolated from a 2002 outbreak displays a unique HBGA pattern (Lewis^A^, Lewis^X^, and A antigens); and another 2002 strain in addition to strains isolated from 2004 and 2005 outbreaks fail to bind efficiently to any HBGA suggesting they may bind other carbohydrates [63,128]. Interestingly, a strain circulating in 2006 displays a similar HBGA binding profile to the 1997 pandemic strain [129]. While norovirus strains display distinct HBGA binding properties, collectively they can infect nearly all individuals due to their high genetic variability [130]. This highlights the highly adaptive nature of noroviruses and the likelihood of a long co-evolution of human noroviruses with their human host.

**Figure 5:**
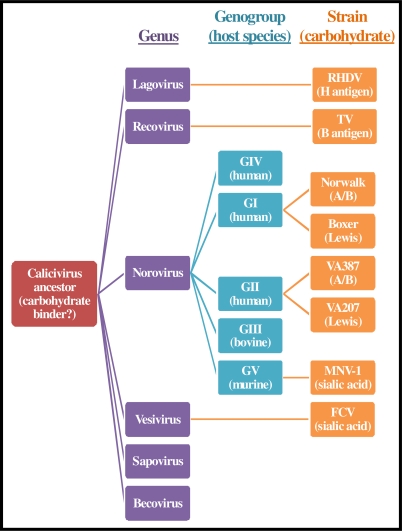
The known carbohydrate binding partners of caliciviruses. At least one member from four of the six calicivirus genera (colored in purple) has been demonstrated to bind a carbohydrate, suggesting that the original calicivirus ancestor was a carbohydrate binder. Similarly, three of the five norovirus genogroups (colored in blue) have been shown to contain members that bind carbohydrates. Two of the three norovirus genogroups containing human members have been shown to specifically bind HBGAs, either A/B/H antigens (A/B) or Lewis antigens (Lewis). Representative virus strains and their known carbohydrate ligands are shown in orange. Data are adapted from *PLoS ONE* **2009**, *4*, e5058.

Several animal caliciviruses also bind HBGAs, including the highly virulent rabbit hemorrhagic disease virus that segregates within the lagovirus genus [131] and the Tulane virus isolated from rhesus macaques that defines the newly proposed recovirus genus [127,132]. Conversely, other animal caliciviruses, including multiple feline calicivirus strains [133] and murine norovirus strains [134], bind sialic acid on permissive cells. Thus, carbohydrate binding appears to be conserved across the *Caliciviridae* family while specific members can bind neutral or negatively charged residues (Figure 5). There are examples of human noroviruses that do not bind HBGAs, including recent GII.4 pandemic strains [125,128]; it has yet to be determined whether these strains instead bind negatively charged sugars similar to feline caliciviruses and murine noroviruses. Evidence supporting this possibility is provided by studies demonstrating binding of GII noroviruses to negatively charged heparan sulfate [135] and sialylated Lewis^x^ carbohydrate [136].

Even though HBGA association is essential for *in vivo* Norwalk infection, it is not sufficient to overcome the block to *in vitro* virus propagation – cell lines expressing HBGAs are resistant to infection [104] and transfection of FUT2 into cells does not facilitate infection [110]. These observations suggest that binding to an as-yet-unidentified entry receptor is also a required event. This is supported by the identification of both JAM-A and sialic acid as receptors for feline calicivirus [133,137]. It is even possible that cells expressing HBGAs are not the true target of norovirus infection. For example, binding to HBGA expressed on the surface of enterocytes may mediate norovirus attachment to the intestinal wall while binding to an alternative receptor on a neighboring cell type (*i.e.,* DCs and Mφs) is required to trigger entry and subsequent replication.

## 5. Molecular Determinants of Norovirus Pathogenesis

Understanding the molecular aspects of viral replication can facilitate the development of rational therapies. In this section, we summarize recent findings of norovirus replication mechanisms that may contribute to norovirus pathogenesis and ultimately lead to the design of novel antiviral strategies.

### 5.1. Norovirus Genomic Structures

**Figure 6:**
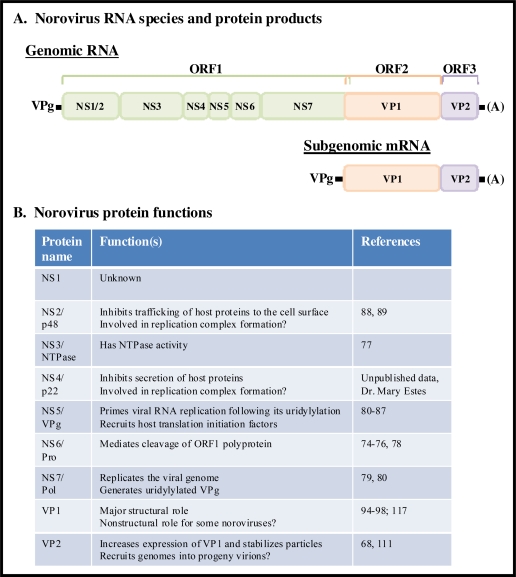
Norovirus genomic organization and protein function. (**A**) Norovirus genomes are comprised of a single linear piece of positive-sense RNA between 7.4–7.7 kb. They contain three open reading frames, one encoding a polyprotein of 7 nonstructural protein products (colored in green), one encoding the major structural capsid protein VP1 (colored in orange), and one encoding the minor structural protein VP2 (colored in purple). There is a short overlap between ORFs 1 and 2. A subgenomic mRNA that is 3’ co-terminal with full-length genomes is produced during norovirus replication. This mRNA acts as a template for the production of structural proteins; similar to genomic RNA, it is covalently linked to VPg at its 5’ end and polyadenylated at its 3’ end. (**B**) Known and hypothesized functions of mature norovirus proteins.

Noroviruses have plus-stranded linear RNA genomes of 7.4–7.7 kb which are typically organized into three open reading frames (ORF1-3) (Figure 6) [138–140]. Murine noroviruses have a predicted fourth ORF overlapping ORF2 but in an alternate reading frame [41]; it has yet to be determined whether this ORF codes for a functional protein. The 5’ proximal region of the norovirus genome encodes all of the nonstructural proteins in a single ORF (ORF1) while the 3’ proximal region, transcribed into a subgenomic message, encodes the major and minor structural proteins in two separate ORFs (ORF2 and ORF3, respectively). Norovirus genomes are thought to be covalently associated with a viral protein called VPg at their 5’ ends and are polyadenlyated at their 3’ ends.

Essential viral genomic structures represent putative antisense drug targets. Moreover, identification of essential RNA elements within the viral genome may facilitate the construction of viruses with a dominant inhibitory effect on the replication of wild-type virus [141]. Recent bioinformatic analyses of calicivirus genomes have identified conserved secondary structures that include: (i) two or more 5’ terminal stem-loops; (ii) a 3’ terminal hairpin; (iii) a stem-loop just upstream of the ORF1/2 junction in the antigenomic strand proposed to be a component of the subgenomic promoter; and (iv) a stem-loop at the 5’ end of the polymerase coding region with a motif characteristic of picornavirus *cre* elements that dictate VPg uridylylation [142,143]. Several of these structures were determined to be critical for the replication of a murine norovirus [142].

### 5.2. Norovirus Nonstructural Proteins

Norovirus ORF1 encodes a large polyprotein that is cleaved into seven mature nonstructural proteins (NS1-NS7) [144–146]. A number of these proteins have defined activities, including an NTPase (**NS3**) [147], a protease (Pro; **NS6**) [148], and an RNA-dependent RNA polymerase (Pol; **NS7**) [149,150]. Noroviruses also encode a VPg (**NS5**) that, based on work with animal caliciviruses, is presumed to be covalently attached to the 5’ ends of viral genomes in place of a typical 5’ cap structure [151–153]. The norovirus VPg can function as a primer in viral RNA replication following its uridylylation [150,154]. However, the norovirus Pol can also initiate RNA polymerization in a primer-independent manner; it has been postulated that it uses uridylylated VPg primer-mediated polymerization to replicate polyadenylated genomes and primer-independent polymerization to copy antigenomes [149,150]. The norovirus VPg also interacts with host translation initiation factors, perhaps to recruit them to the 5’ end of the RNA genome for translation initiation [155–157].

A number of the norovirus nonstructural proteins do not share significant sequence homology to proteins of known function, including NS1, NS2, and NS4 [4], and their functions in viral replication are only beginning to be unraveled. While there is no information available on the role played by the most amino-terminal protein **NS1** in norovirus replication**, NS2** (also referred to as p48 and the N terminal protein) has been shown to co-localize with the Golgi complex in transfected cells and induce rearrangement of the Golgi membrane [158]. It also interacts with a host protein involved in regulating vesicle transport and can inhibit cell surface expression of proteins [159]. Expression of **NS4** (also referred to as p22 and the 3A-like protein) in transfected cells has recently been shown to induce Golgi disruption. Moreover, NS4 contains a novel ER export signal that results in aberrant trafficking of COPII-coated vesicles and ultimately in inhibition of host protein secretion (Sharp TM, Guix S, Katayama K, Crawford SE and Estes MK, manuscript submitted). Redistribution of COPII-coated vesicles was not observed during MNV-1 infection of RAW264.7 cells [160], suggesting that human and murine noroviruses may differentially affect secretory pathways. Overall, available data suggest that norovirus NS2 and NS4 proteins both contribute to viral replication complex formation on intracellular membranes including that of the Golgi apparatus. This is supported by work with MNV-1, demonstrating that all nonstructural proteins localize to the ER, Golgi bodies, and early endosomes, but not to late endosomes or lysosomes [160]. One can postulate that NS2 and NS4 disrupt intracellular host protein trafficking, which may play an important role in the pathogenesis of norovirus infections by blocking surface expression (*i.e.,* MHC molecules) and secretion (*i.e.,* cytokines) of immune mediators in infected cells.

Any of the essential activities encoded by norovirus nonstructural proteins could potentially be targeted with antiviral compounds for intervention. In addition, immunotherapeutic strategies based on enhancing the host cell’s normal antiviral mechanisms may be engineered to disrupt the function of one or more norovirus nonstructural proteins. For example, during MNV-1 infection of permissive cells, translation of nonstructural proteins can be inhibited by both type I and type II interferon responses [161]. A host innate immune mediator induced by interferon may thus block required interactions between norovirus VPg and translation initiation factors, representing a candidate immunotherapeutic to treat norovirus infections. Both type I and type II interferon also inhibit the replication of a human norovirus expressed from a replicon in transfected cells [115,162]; it has yet to be determined whether these responses target norovirus translation as they do in the murine system.

### 5.3. Norovirus Structural Proteins

Noroviruses are nonenveloped icosahedral viruses between 27–30 nm in diameter. Norovirus ORF2 encodes the major structural protein called VP1 or capsid. Virions contain 180 copies, or 90 dimers, of VP1 that assemble into icosahedral particles exhibiting T = 3 symmetry [163–167]. Each capsid dimer forms an arch-like protrusion extending from the internal shell of the particle. The capsid protein itself can be divided into a conserved internal shell domain (S) and a more variable protruding domain (P) that forms the arch-like protrusions. The P domain can be further subdivided into P1 and P2 domains, with P1 forming the sides of the arches and P2 located at the exposed tops of the arches. Expression of VP1 independent of other viral components results in the self-assembly of virus-like particles (VLPs) [168], and expression of solely the P domain of VP1 results in the formation of P dimers as well as P particles composed of 12 P dimers [169]. These structures retain the binding properties of native norovirus virions, at least in terms of carbohydrate association, and have been extremely useful in binding and antigenicity studies in the absence of a cell culture system [170]. Consistent with its exposed location on the virion surface, the P2 domain of VP1 comprises a hypervariable region that is suspected to contain receptor binding and antigenic sites. Indeed, numerous structural and mutagenesis studies of VLPs and P domain structures have confirmed that the binding interface for HBGA association with norovirus particles resides in the P2 domain of capsid [169,171–175]. Furthermore, monoclonal antibodies have been demonstrated to recognize conformational epitopes located in the P2 domain [176,177]. Identifying critical determinants of norovirus receptor binding and antigenicity may be instrumental in the design of effective therapeutics and vaccines. Interestingly, a single amino acid change in the P2 domain of a murine norovirus has been associated with its *in vivo* attenuation [103,178].

Norovirus ORF3 encodes a minor structural protein called VP2 that is present in only 1–2 copies per virion [138,179]. VP2 is small, basic, and quite divergent in both size and sequence between viruses within this family. Its function in viral replication is currently undefined but there is evidence that it increases the expression level of VP1 and that it stabilizes VLPs [180]. The basic charge of VP2 suggests that it may function in encapsidation of the viral genome.

Both VP1 and VP2 are translated from a subgenomic message that is polyadenylated and, based on work with feline caliciviruses [152], is presumed to be covalently attached to VPg at its 5’ end. While expression of VP1 is presumed to occur via VPg-directed translation initiation (similar to expression of nonstructural proteins from genomic RNA), the mechanism for translation initiation of the downstream VP2 protein was unclear until recently. There is now evidence from several animal caliciviruses that translation of VP2 proceeds via a translation termination re-initiation (TTR) mechanism. In this process, posttermination ribosomes from VP1 translational events remain associated with subgenomic RNA through interactions with termination upstream ribosomal binding sites (TURBSs) in the 3’ proximal end of VP1, facilitating recruitment of translation initiation factors to the 5’ end of VP2 [181–184]. At least one of the TURBS is conserved across the *Caliciviridae* family, suggesting that noroviruses rely on the same mechanism for VP2 expression [181]. A recent study also suggests that TTR is used by at least one norovirus to express VP1 independent of subgenomic message production [185]; while this most likely contributes minimal amounts of capsid compared to the levels produced from translation of subgenomic RNA, the authors speculate that VP1 may play an additional nonstructural role(s) in the viral replication cycle.

## 6. Norovirus Immunity and Vaccination

### 6.1. Immunity

Immunity to noroviruses is complicated by the heterogeneous responses of the human population and the transient nature of immunity in some individuals. These atypical responses are best highlighted by early studies in which volunteers were repeatedly exposed to homologous virus inoculum comprised of filtered stool from norovirus outbreak patients [5,6]. In these studies, several patterns of susceptibility were noted – a proportion of volunteers were resistant to primary and subsequent infections, and a proportion of individuals were equally susceptible to primary and secondary infections when there was at least a six-month interval between challenges. One possible explanation for the continued susceptibility of a subset of individuals is that they fail to generate a virus-specific immune response upon norovirus infection. However, data gathered from many human volunteer challenge studies and natural norovirus outbreaks conclusively show that people do develop a virus-specific antibody response and that the presence of this response does not correlate with protection [5,6,93,123,186–189]. Specifically, virus-specific serum IgG is induced and persists for months following infection, while serum IgA and IgM responses are more short-lived. Mucosal IgA is induced as well but its duration has not been determined [190]. Because there is no cell culture system for human noroviruses, it has not been possible to determine whether norovirus-specific antibodies are neutralizing. However, a surrogate assay based on inhibition of VLP binding to HBGA ligands has been used to demonstrate that virus-specific serum antibodies generated during experimental and natural infections neutralize carbohydrate binding [128,129,191,192]. Moreover, traditional neutralization assays are possible for murine noroviruses because they are cultivable. MNV-1 infection of mice induces neutralizing serum antibodies [103] and one MNV-1-neutralizing monoclonal antibody has been shown to bind each of the 180 hypervariable P2 domains of native MNV-1 virions without inducing a major conformational change, suggesting that neutralization in this case is mediated by abrogation of cellular attachment [167]. Nevertheless, the duration of norovirus antibody responses has not been clearly defined, so it remains possible that waning humoral immunity is related to the susceptibility of some previously exposed individuals to repeat norovirus infections.

While there have been no direct studies of T cell responses to human norovirus infection, surrogate cytokine studies suggest that virus-specific T cells are also induced in infected individuals. In one study, peripheral blood mononuclear cells (PBMCs) were collected from norovirus-infected volunteers at pre-challenge and post-challenge time points, incubated with norovirus VLPs, and secretion of cytokines from the PBMCs was measured [193]. The authors noted increases in the Th1 cytokines IFNγ and IL-2, as well as the Th2 cytokine IL-5. Notably though, similar increases in cytokine levels were observed when pre-challenge and post-challenge PBMCs from uninfected individuals were compared to each other, presumably indicating that controls in this study were previously exposed to natural norovirus infection. This finding highlights the difficulty of studying primary immune responses to human noroviruses where infection histories are impossible to discern. Recent studies in the gnotobiotic pig and calf models also report moderate increases in several Th1 and Th2 cytokines as well as interferon in the serum and intestinal contents of infected animals, suggestive of a T cell response upon primary challenge [74,194]. Overall, the available information on secondary human norovirus infections supports an atypical pattern of immunity in which some previously exposed individuals are susceptible to repeat infections even in the face of virus-specific memory immune responses. Another subset of individuals is resistant to infection, presumably due to genetic resistance (*i.e.,* nonsecretors) or preexisting immunity.

**Figure 7:**
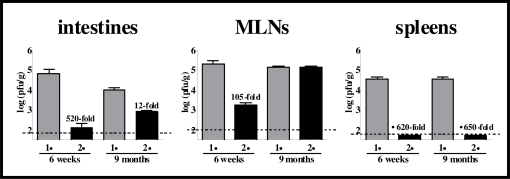
Mucosal norovirus immunity wanes over time. Groups of 129SvEv mice (5–14 mice per group) were mock-infected (1°) or inoculated perorally with 10^4^ pfu MNV-1.CW3 (2°). Six weeks or nine months later, all mice were infected with 10^7^ pfu MNV-1.CW3. One day after secondary challenge, animals were perfused, organs were harvested, and viral burden was determined by plaque assay. The data for all mice in each group are averaged. Limits of detection are indicated by dashed lines. Fold-reductions for titers in mice receiving secondary challenge compared to those in mice receiving primary challenge are listed above the black bars.

In the early volunteer challenge studies, it was noted that susceptible individuals were protected from disease when re-challenged soon after secondary exposure (<6 months) in contrast to a more distant re-challenge (>6 months) [5,6], suggesting that short-term norovirus immunity is elicited whereas long-term immunity is not. Studies of murine norovirus infection lend support to this explanation – a primary MNV-1 exposure results in reduced virus loads upon re-challenge when the interval between primary and secondary infections is short (Figure 7; 6-week challenge), although it is important to note that this magnitude of protection is insufficient to protect the host from disease [88]. While protective peripheral immunity in this setting is maintained over time, protective mucosal responses wane (Figure 7, 9-month challenge; Karst laboratory, unpublished data), indicating that memory immune responses at mucosal sites are not efficiently maintained. There is also evidence from the murine model of norovirus infection that repeated norovirus exposure boosts the long-term immune response – repeated exposure to high-dose MNV-1 elicits long-lasting protective immunity [99], whereas single high-dose infection does not [88]. The immunity elicited by repeated exposure to MNV-1 does appear to be lasting [99]. Interestingly, studies of murine norovirus infection also signify that lower doses of virus elicit stronger memory immune responses than high doses [88]. It has yet to be determined whether virus dose similarly displays an inverse correlation to protection upon human norovirus infection.

**Figure 8:**
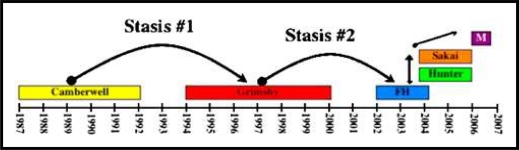
Epochal evolution of the GII.4 noroviruses. Early GII.4 norovirus strains (Camberwell and Grimsby) persisted for long periods and were followed by shorter periods of stasis before being replaced by a new dominant strain. The evolution of GII.4 viruses appears to have become much more rapid in recent years, with pandemic strains being replaced by a new dominant strain in 1–2 years. FH, Farmington Hills; M, Minerva.

In apparent contradiction to the results of early human volunteer studies, some investigators have recently suggested that noroviruses can elicit herd immunity [62,123,129,193]. This supposition is based on the epochal pattern of GII.4 pandemics, with emergence of a dominant virus strain for a 1–2 year period followed by a period of relative GII.4 quiescence prior to emergence of a new dominant strain (Figure 8) [62]. The emergence of new pandemic GII.4 strains is postulated to result from (i) antigenic drift-mediated altered carbohydrate usage and (ii) altered antigenicity facilitating virus escape of herd immunity [128,195]. Consistent with this, only a minimal number of amino acid changes in the capsid P domain are required to alter HBGA usage, enabling norovirus strains great flexibility in evolving to infect new populations [195]. It has also been reported in a recent human norovirus challenge study that a subset of secretor-positive volunteers were resistant to Norwalk virus infection, and these individuals displayed a modest increase in norovirus-specific salivary IgA early after infection [123]. While interesting, interpretation of these studies is complicated by several factors: the previous exposure histories of the subjects are unknown, and it is not possible to distinguish between short- and long-term immunity. Moreover, these studies do not offer an explanation for the observation that some individuals are susceptible to repeated infections with homologous virus [5,6].

One explanation that would be inclusive of all norovirus immunity observations to date is that short-term herd immunity drives the evolution of emerging norovirus strains, but this immunity wanes over time such that genetically susceptible individuals can be repeatedly infected with homologous virus over the long-term. Studies of GII.4 carbohydrate usage may support this idea – while 2002–2005 strains display distinct binding profiles compared to earlier 1974–1997 strains, the binding profile of the more recent 2006 GII.4 strain is similar to those of 1974–1997 strains [63,195].

### 6.2. Candidate Norovirus Vaccination Strategies

Current efforts to design human norovirus vaccines focus on expression of the norovirus capsid protein in various vectors, resulting in self-assembly of the capsid protein into immunogenic VLPs [4]. Such vaccine candidates elicit both serum IgG and mucosal IgA responses, but the duration of these responses and their protective potential have not been determined. A Phase I safety trial has been completed for a dry powder norovirus VLP vaccine delivered intranasally in conjunction with a TLR4 agonist adjuvant; the vaccine was determined to be immunogenic and was not associated with adverse side effects (http://www.ligocyte.com/downloads/Noro.pdf). Live virus challenge studies are now underway. While challenge studies following exposure to human norovirus VLPs have not been reported, results from animal models are encouraging: (i) intranasal inoculation of calves with bovine norovirus VLPs + adjuvant induces both mucosal and systemic humoral immunity, although protection from subsequent challenge is only partial and viral shedding is not prevented [196]; (ii) repeated mucosal exposure to HS66 VLPs + adjuvant elicits immune responses and protection from live HS66 in a majority of vaccinated gnotobiotic pigs [197]; (iii) footpad inoculation of mice with murine norovirus VLPs + adjuvant induces humoral immune responses and reduced virus loads, although protection from disease was not assessed in this study [198]. Additional work with murine norovirus suggests that a broad adaptive immune response including B cells, CD4 T cells, and CD8 T cells, is critical for full protection [99].

While available information on norovirus VLP-based vaccines is promising, several potential obstacles require serious consideration. Feline calicivirus vaccines, either modified live vaccines or inactivated vaccines given with adjuvants, have been used for many years [95]. These vaccines induce protective immunity in cats in terms of clinical disease prevention; however, they do not prevent infection or persistence. In such a scenario, the presence of non-neutralizing immunity could actually induce the emergence of virulent strains [199]. To this end, multiple virulent feline calicivirus isolates have recently emerged that cause systemic disease associated with high mortality; vaccinated cats have been prominently affected in these outbreaks [200,201]. These data highlight the potential necessity of designing norovirus vaccination strategies that target both clinical disease and prolonged infection. Other major obstacles in designing norovirus vaccines are the genetic variability within this virus family and the replacement of pandemic strains in short time intervals (see Figure 8). These factors may require a continually evolving vaccine preparation similar to the one currently in place for influenza viruses. Moreover, multiple norovirus strains may need to be included in this preparation to elicit effective immunity to each genogroup. A final possible hindrance to a successful norovirus vaccine is the inability of natural infection to elicit lasting protection in all individuals. It will be critical to delineate the basis for this lack of immunity and to determine whether a similar phenomenon occurs upon exposure to candidate norovirus vaccines.

## 7. Conclusions

Recent advances in the norovirus field have greatly facilitated progress in our understanding of how this emerging RNA virus family causes disease and persists worldwide. These advances include the identification and structural characterization of HBGAs as receptors, the development of animal models and a murine norovirus cell culture system, and the design of norovirus infectious clones and replicons. While much progress has been made in the human norovirus field, it is still limited by the lack of an efficient cell culture model. Designing efficacious norovirus vaccines and therapies with broad spectrum activity across the family also remains a major challenge due to several factors: (1) the extreme genetic variability within the norovirus family and within genogroups; (2) the rapid evolution of antigenically dissimilar pandemic GII.4 norovirus strains; and (3) the lack of lasting immunity upon natural exposure to noroviruses in at least a proportion of the population. Studies in the murine norovirus model suggest that immunity to natural infection does indeed wane over time but can be enhanced by repeat exposure. Moreover, the finding that the HBGA binding interface is conserved within a norovirus genogroup may facilitate the development of novel therapeutic strategies that target all members of a specific genogroup. As we continue to expand our understanding of norovirus replication strategies using newly developed *in vitro* models, additional novel targets for antiviral therapies will undoubtedly be revealed.
